# A comprehensive description of oocyte developmental stages in Pacific halibut, *Hippoglossus stenolepis*

**DOI:** 10.1111/jfb.14551

**Published:** 2020-10-05

**Authors:** Teresa Fish, Nathan Wolf, Bradley P. Harris, Josep V. Planas

**Affiliations:** ^1^ Fisheries, Aquatic Science and Technology Laboratory Alaska Pacific University Anchorage Alaska 99508 USA; ^2^ International Pacific Halibut Commission Seattle Washington USA

**Keywords:** developmental stage, histology, maturity, oocyte, Pacific halibut, reproduction

## Abstract

Accurate characterization of oocyte development is essential to understanding foundational aspects of reproductive biology and successful management of Pacific halibut (*Hippoglossus stenolepis*). Here this study provides complete histological descriptions for eight oocyte developmental stages in addition to postovulatory follicles and demonstrates the potential for oocyte size frequency distribution to act as a proxy for ovarian developmental stage and future maturity assessments. Importantly, it provides the first histological evidence that Pacific halibut have a group‐synchronous ovarian developmental pattern with determinate fecundity and support for their batch‐spawning strategy.

Understanding the species‐specific components of fish reproductive biology (*e.g*., age at maturity, fecundity, spawning strategy) is foundational for effective stock management. These indices vary by species (Kennedy *et al*., 2014; Núñez *et al*., 2015; TenBrink & Wilderbuer, 2015) and can dramatically alter our perception of stock status (Morgan, 2008). This is especially true for long‐lived fish species, such as Pacific halibut (*Hippoglossus stenolepis*), as lifetime contributions to stock recruitment continue for many seasons after reproductive maturity is reached. For example, changes in reproductive performance, as deduced from female maturity estimates, exert a strong influence on spawning biomass estimates and, consequently, on the stock assessments of the Pacific halibut (Stewart & Hicks, 2020).

Currently, assessments of female Pacific halibut reproductive maturity involve visual macroscopic inspection of ovaries in the field (Stewart & Hicks, 2020). While convenient, this approach has yet to be corroborated by more definitive analysis methods and lacks the specificity required to provide information on many species‐specific components of reproductive biology. As accurate characterizations of reproductive development are essential to fisheries management, an evaluation of the reliability of the current macroscopic staging methods using more precise assessment techniques is of utmost importance.

Histological analysis of oocyte developmental stages represents an important initial step to evaluating current maturity assessment methods (West, 1990). Moreover, when providing oocyte size‐frequency distributions, histological analyses may also offer alternative methods for characterizing fish reproductive phases and offer additional information on reproductive parameters, such as fecundity type and spawning pattern. Histological examinations have successfully characterized ovarian development in many flatfishes, including California halibut *Paralichthys californicus* (Lesyna & Barnes, 2016) and Atlantic halibut *Hippoglossus hippoglossus* (Neilson *et al*., 1993). Furthermore, framing this characterization using universally descriptive terminology for teleost oogenesis (Grier *et al*., 2009) will facilitate future comparative examinations (Brown‐Peterson *et al*., 2011).

Pacific halibut represent an important economic and cultural resource in the Gulf of Alaska and the rest of the northeastern Pacific Ocean. In this region, spawning occurs between November and March along the continental slope and in depressions on the continental shelf (St‐Pierre, 1984). Spawning is generally thought to occur annually after fish reach reproductive maturity (Stewart & Hicks, 2020; St‐Pierre, 1984; Thompson, 1914), but this assumption has received mixed support in the literature (Bell, 1981; Novikov, 1964; Seitz *et al*., 2005; Vernidub, 1936). Early investigations of Pacific halibut oocyte size (Kolloen, 1934; Thompson, 1914, 1916) documented a developing cohort of oocytes within the ovary immediately after spawning, thus supporting the premise of annual spawning. Nonetheless, a detailed histological characterization of ovarian development in female Pacific halibut to be used in histology‐based reproductive phase maturity classification has not been conducted to date.

Here, this study presents comprehensive histological descriptions of oocyte developmental stages and documents postovulatory follicles (POFs) in Pacific halibut. In addition, it details the range of oocyte diameters among developmental stages and explores differences in the size‐frequency distributions of oocytes in ovarian tissue at different developmental stages to investigate the relation between oocyte size and oocyte developmental stages. This work provides the most precise assessment of developmental stage for the species to date, documents the spawning strategy and offers a foundation for more specific assessments of Pacific halibut reproductive biology in the future.

To conduct this study, the authors collected approximately 30 female Pacific halibut each month from September 2017 through August 2018 (*n* = 356) from the Portlock region in the Gulf of Alaska using longline fishing vessels specifically chartered for sampling. Individual sampling trips occurred over 1–4 day periods. The authors focused collection efforts on larger (≥ 90 cm) individuals to increase the probability of sampling postpubescent fish (Clark *et al*., 1999; Loher & Seitz, 2008). Once fish were on‐board the fishing vessel, approximately 1 cm^3^ tissue was excised from the central area of the ovary and fixed in 10 ml of 10% buffered formalin from each fish. Ovarian tissue samples were sent to an independent laboratory to be processed for histology where two series of 4 μm thick Paraffin sections, separated by approximately 500 μm, were mounted on two slides and stained with haematoxylin and eosin.

Ovarian follicles in mounted and stained ovarian tissue sections were examined visually under 1×–10× magnification (Leica DM LB2 and Leica M80 microscopes, Leica Camera Inc., Allendale, NJ, USA). Oocyte developmental stages were categorized following the universal oocyte stages described by Brown‐Peterson *et al*. (2011) with the exception that the germinal vesicle breakdown and hydration stages were combined into a single periovulatory stage (PO; Table 1). In addition, the early growth phases detailed by Grier *et al*. (2009) are used to describe primary growth (PG) oocytes. Individual female developmental stages were assigned based on the most advanced oocyte developmental stage present in the ovarian tissue (Table 1).

**TABLE 1:** Description of oocyte developmental stages of Pacific halibut, *Hippoglossus stenolepis*, associated growth phases (modified from Brown‐Peterson *et al*., 2011 and Grier *et al*., 2009), and postovulatory follicles (POFs)

Pacific halibut oocyte histology					Oocyte diameters (μm)	
Growth phase (acronym)	Developmental stage (acronym)	Description	Photo	Sample size	Mean ± S.D.	Range (min–max)
Primary growth (PG)	One nucleolus (PGon)	Oocytes are small, angular and compact with a single large nucleolus. Cytoplasm granules stain dark purple.	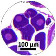	51	116 ± 89	36–381
	Perinucleolar (PGpn)	Oocytes are larger and rounder than PGon. Nuclei develop and flatten around the nucleus. Cytoplasm granules stain light purple.	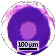	55	235 ± 92	103–479
	Cortical alveolar (CA)	First cortical alveoli appear as white stain in the periphery of the oocyte.	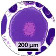	237	445 ± 80	195–664
Secondary growth (SG)	Primary vitellogenesis (Vtg1)	Yolk globules first appear at the periphery, stain pink and fill inwards occupying up to one‐third of the cytoplasm.	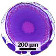	663	544 ± 69	362–750
	Secondary vitellogenesis (Vtg2)	Yolk globules transition from only the periphery of the ooplasm and fill inwards to the nucleus.	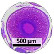	341	686 ± 91	465–910
	Tertiary vitellogenesis (Vtg3)	Yolk globules completely fill the ooplasm to the central nucleus and coalesce into larger yolk globules.	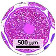	500	1171 ± 216	706–1644
Oocyte maturation (OM)	Germinal vesicle migration (GVM)	The nucleus begins to migrate through a cytoplasm fully filled with large yolk globules.	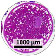	302	1271 ± 257	811–1769
	Periovulatory (PO)	Nucleus no longer visible and the yolk globules coalesce into a central yolk mass. Oocyte is still within the follicle wall.	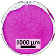	54	2037 ± 270	1600–2811
	Postovulatory follicle (POF)	Collapsed empty follicle wall remaining after a periovulatory oocyte is expelled.	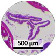			

To assess potential differences in oocyte diameter among oocyte developmental stages, 3–5 of the most advanced stage oocytes (*i.e*., largest) from each fish were photographed at 1×–10× magnification using a microscope‐mounted camera (Zeiss Axiocam ERc 5s or Leica IC80 HD; Carl Zeiss Microscopy LLC, White Plains, NY, USA and Leica Camera Inc., Allendale, NJ, USA, respectively) and a minimum of 50 oocytes sectioned through the middle of the nucleus from each developmental stage were measured (Table 1). Area measurements (μm^2^) of photographed ovarian follicles were conducted using ImagePro Premier 9.1 (Media Cybernetics Inc., Rockville, MD, USA) by tracing the perimeter of the oocyte and extracting the oocyte area. All measured areas were converted to mean diameters for further analysis in ImagePro Premier 9.1 by calculating the mean length of diameters measured at 2 degree intervals passing through the traced oocyte centroid. Differences in the mean rank oocyte diameter among developmental stages were analysed using the Kruskal–Wallis rank sum test, followed by Dunn's *post hoc* test. All statistical analyses were conducted in R version 3.5.2.

Potential differences in the size distribution of oocytes present in fish from different developmental stages were examined following methods detailed in Guzmán *et al*. (2017). Briefly, a 161 mm^2^ image of mounted ovarian tissue was scanned at 9600 dpi (Epson Perfection V800 photo scanner; Epson US, Hillsboro, OR, USA) from a sub‐sample of five fish of each observed female developmental stage, from cortical alveolar (CA) to PO, and from the three fish classified in the PG perinucleolar (PGpn) stage (Table 1). Using these scanned images, the areas of all oocytes sectioned through the nucleus within a 9, 25 or 64 mm^2^ region of interest were measured. In all cases, the smallest region in which at least 18 oocytes could be measured was used. Area measurements and diameter conversions were performed using the methods described earlier. To facilitate the visualization of the results, oocyte areas of each developmental stage were natural log (ln) adjusted, then summed across 50 μm diameter bins. Results are presented as proportional occupancy of each ln‐adjusted oocyte developmental stage.

The present study provides histological descriptions of eight oocyte developmental stages [PG one nucleolus (PGon), PGpn, CA, primary, secondary and tertiary vitellogenesis (Vtg1‐3, respectively), germinal vesicle migration (GVM) and PO] as well as POFs in Pacific halibut (Table 1). The results represent the first description of oocyte development stages in Pacific halibut. Oocyte diameters were measured from each of the eight oocyte developmental stages and ranged from 36 μm for the smallest oocyte in the PGon stage to 2811 μm for the largest oocyte in the PO stage (Table 1). In all cases, mean oocyte diameters increased with progression through developmental stages and significant differences in mean rank diameters were observed among all developmental stages (Kruskal–Wallis rank sum test: χ^2^ = 1906.3, *df* = 7, *P* < 0.001; Dunn's *post hoc* test, *P* < 0.025) except between PGon and PGpn and between Vtg3 and GVM (Figure 1a). No atretic oocytes were identified in the present study. The presence of POFs and atretic oocytes will be further investigated in a future study evaluating seasonal or temporal changes in maturity in female Pacific halibut.

**FIGURE 1:**
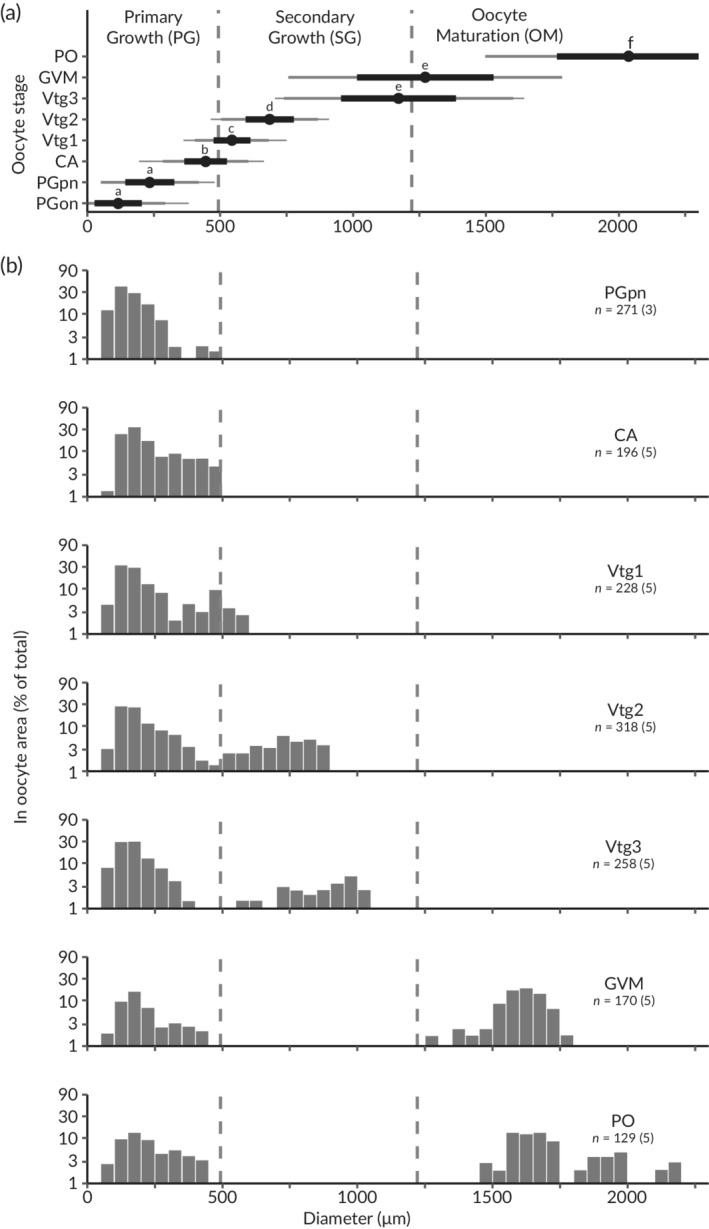
Description of changes in Pacific halibut mean oocyte diameters with (a) developmental stage and (b) oocyte size distribution with individual fish developmental stage. Vertical broken lines separate the growth stages at the midpoint between mean oocyte developmental stage diameters. In (a), mean oocyte diameters are indicated by black circles, one‐ and two standard deviations are indicated by wide black and grey bars, respectively; and minimum and maximum values are indicated by narrow grey bars, with different letters (a–f) indicating statistical significance (*P* < 0.025) among groups. In (b) the distribution of oocyte diameters is represented as the proportional occupancy of ln(oocyte area) for each of the observed female developmental stages (defined as the most advanced stage of oocyte present in the sample population; PGpn, PGca, *etc*.), with *n* indicating the total number of oocytes measured from a sub‐set of females (number of individual fish shown in parentheses)

Explorations of oocyte size distributions in fish at different female developmental stages showed that Pacific halibut follow a pattern typical of fish species with group‐synchronous ovarian development with determinate fecundity (Ganias, 2013; Lubzens *et al*., 2010). Fish in early developmental stages displayed unimodal distributions of oocyte diameters, which became increasingly right‐skewed from PGpn to CA (Figure 1b). This mode – generally up to 500 μm in diameter – shares similar morphological characteristics to corresponding PG oocytes described in other fish species (Grier *et al*., 2009; Selman *et al*., 1993) and was present in all female developmental stages (Figure 1b). Fish in more advanced developmental stages displayed bimodal oocyte size distributions, with increasing separation between the two modes with progressing developmental stage (Figure 1b). This second mode developed as Vtg1 oocytes further separated in size from the previtellogenic cohort at the Vtg2 developmental stage (Figure 1b). At the Vtg3 developmental stage, a hiatus was present between the two modes, effectively separating the previtellogenic mode (oocytes <350 μm in diameter) and the larger or leading cohort of vitellogenic oocytes (> 500 μm in diameter). At the GVM and PO developmental stages, oocytes in the leading cohort continued to increase in size (> 1200 μm in diameter) up until hydration (*c*. 2000 μm in average). The selective recruitment of oocytes from early into later (*i.e*., GVM and PO) female developmental stages, as shown by the gradual transition from unimodal to bimodal size distributions leading to two different populations of oocytes in the more advanced stages of ovarian development, is evidence for a group‐synchronous ovarian developmental pattern with determinate fecundity in this species (Ganias, 2013; Lubzens *et al*., 2010). Furthermore, although the most advanced oocytes in the GVM developmental stage represented a unique, leading mode, the leading oocytes in the PO developmental stage showed a larger range of sizes that included GVM and PO oocytes at different steps of hydration (Figure 1b). These observations suggest that, despite the lack of information of the temporal progression of these events and the relatively small sample size in the PO developmental stage, GVM oocytes may be recruited for final maturation, hydration and subsequent ovulation in batches, supporting the notion that Pacific halibut, like its Atlantic congener (Haug & Gulliksen, 1988), is a batch spawner, as suggested by a previous report of the spawning behaviour of one tagged Pacific halibut female (Seitz *et al*., 2005).

The histological description of oocyte developmental stages produced by this work provides an important and necessary framework for characterizing reproductive phase and allows for future explorations of reproductive development in Pacific halibut. The authors propose future research to evaluate seasonal changes in ovarian developmental stages, as defined here, as well as reproductive phases by investigating POFs and atresia observed in conjunction with the developmental stages. After a thorough investigation of reproductive phases in Pacific halibut, the authors further recommend future research in the area of assessing the accuracy of current macroscopic reproductive maturity staging methods, with special attention to the potential identification of skip‐spawning females and their assignment to particular macroscopic maturity stages. The authors believe that their results on oocyte menstruation and size‐frequency distributions presented here will be important to explore potentially time‐ and cost‐efficient alternatives to histological oocyte evaluations for reproductive assessments in this commercially important species. Oocyte measurement methods have previously been demonstrated as effective reproductive phase indicators in Atlantic halibut (Neilson *et al*., 1993), and newer methods (Friedland *et al*., 2005; Thorsen & Kjesbu, 2001; Witthames *et al*., 2009) with improved detection, cost and efficiency could potentially be applied to Pacific halibut. Although fecundity at size or age of Pacific halibut is a knowledge gap, it should be noted that although mean oocyte diameter has been linked to oocyte density for use in calculating fecundity (Thorsen & Kjesbu, 2001), the analysis would require additional oocyte diameter explorations to calibrate whole mount oocytes with the histology‐derived oocyte diameters presented here.

## CONFLICT OF INTEREST

All authors declare that they have no conflict of interest.

## AUTHOR CONTRIBUTIONS

T.F. contributed to the study design, collected the samples, analysed and interpreted the results, and was the primary manuscript author. N.W. assisted with analysis, interpretation and presentation of the results and assisted T.F. in writing the manuscript. B.P.H. assisted with the presentation of the results and edited the manuscript. J.V.P. conceived the study, supervised data collection, analysis and interpretation, and edited the manuscript. All authors read and approved the final manuscript.

## COMPLIANCE WITH ETHICAL STANDARDS

Pacific halibut collections were conducted under a letter of acknowledgement from National Oceanographic and Atmospheric Administration (NOAA) Fisheries and biological samples were obtained following guidelines for the euthanasia of finfish from the American Veterinary Medical Association (AVMA, 2020).
